# The complexity of tumor shape, spiculatedness, correlates with tumor radiomic shape features

**DOI:** 10.1038/s41598-019-40437-5

**Published:** 2019-03-13

**Authors:** Elaine Johanna Limkin, Sylvain Reuzé, Alexandre Carré, Roger Sun, Antoine Schernberg, Anthony Alexis, Eric Deutsch, Charles Ferté, Charlotte Robert

**Affiliations:** 1 Gustave Roussy, Université Paris-Saclay, Department of Radiotherapy, F-94805 Villejuif, France; 2 U1030 Radiothérapie Moléculaire, Université Paris-Sud, Gustave Roussy, Inserm, Université Paris-Saclay, 94800 Villejuif, France; 3 Université Paris Sud, Université Paris-Saclay, F-94270 Le Kremlin-Bicêtre, France; 4 Gustave Roussy, Université Paris-Saclay, Department of Medical Physics, F-94805 Villejuif, France; 5 Gustave Roussy, Université Paris-Saclay, Department of Oncology, F-94805 Villejuif, France

## Abstract

Radiomics extracts high-throughput quantitative data from medical images to contribute to precision medicine. Radiomic shape features have been shown to correlate with patient outcomes. However, how radiomic shape features vary in function of tumor complexity and tumor volume, as well as with method used for meshing and voxel resampling, remains unknown. The aims of this study are to create tumor models with varying degrees of complexity, or spiculatedness, and evaluate their relationship with quantitatively extracted shape features. Twenty-eight tumor models were mathematically created using spherical harmonics with the spiculatedness degree *d* being increased by increments of 3 (*d* = 11 to *d* = 92). Models were 3D printed with identical bases of 5 cm, imaged with a CT scanner with two different slice thicknesses, and semi-automatically delineated. Resampling of the resulting masks on a 1 × 1 × 1 mm^3^ grid was performed, and the voxel size of each model was then calculated to eliminate volume differences. Four MATLAB-based algorithms (isosurface (M1), isosurface filter (M2), isosurface remeshing (M3), and boundary (M4)) were used to extract nine 3D features (Volume, Surface area, Surface-to-volume, Compactness1, Compactness2, Compactness3, Spherical Disproportion, Sphericity and Fractional Concavity). To quantify the impact of 3D printing, acquisition, segmentation and meshing, features were computed directly from the stereolithography (STL) file format that was used for 3D printing, and compared to those computed. Changes in feature values between 0.6 and 2 mm slice acquisitions were also compared. Spearman’s rank-order correlation coefficients were computed to determine the relationship of each shape feature with spiculatedness for each of the four meshing algorithms. Percent changes were calculated between shape features extracted from the original and resampled contoured images to evaluate the influence of spatial resampling. Finally, the percent change in shape features when the volume was changed from 25% to 150% of their original volume was quantified for three distinct tumor models and compared to the percent change observed when modifying the spiculatedness of the model from *d* = 11 to *d* = 92. Values extracted using isosurface remeshing method are the closest to the STL reference ones, with mean differences less than 10.8% (Compactness2) for all features. Seven of the eight features had strong significant correlations with tumor model complexity irrespective of the meshing algorithm (r > 0.98, p < 10^-4^), with fractional concavity having the lowest correlation coefficient (r = 0.83, p < 10^-4^, M2). Comparisons of features extracted from the 0.6 and 2 mm slice thicknesses showed that mean differences were from 2.1% (Compactness3) to 12.7% (Compactness2) for the isosurface remeshing method. Resampling on a 1 × 1 × 1 mm^3^ grid resulted in between 1.3% (Compactness3) to 9.5% (Fractional Concavity) mean changes in feature values. Compactness2, Compactness3, Spherical Disproportion, Sphericity and Fractional Concavity were the features least affected by volume changes. Compactness1 had a 90.4% change with volume, which was greater than the change between the least and most spiculated models. This is the first methodological study that directly demonstrates the relationship of tumor spiculatedness with radiomic shape features, that also produced 3D tumor models, which may serve as reference phantoms for future radiomic studies. Surface Area, Surface-to-volume, and Spherical Disproportion had direct relationships with spiculatedness while the three formulas for Compactness, Sphericity and Fractional Concavity had inverse relationships. The features Compactness2, Compactness3, Spherical Disproportion, and Sphericity should be prioritized as these have minimal variations with volume changes, slice thickness and resampling.

## Introduction

Radiomics is the process of extracting high-throughput quantitative data from medical images to contribute to current paradigms in disease diagnosis, staging, management and prognostication^1–3^. In recent years, there has been a rapid increase in publications on radiomics, but their routine utilization in the clinics is still to be achieved.

In complement to textural features, shape features are often extracted in radiomic analysis to describe tumor aggressiveness. Using CT (Computed Tomography) or MR (Magnetic Resonance) images, some tumors are described as spiculated or having ‘ill-defined borders’, which indicates potential to spread to contiguous structures and association with advanced stages^4^. On the contrary, less aggressive and benign tumors frequently have well-defined margins^5^. Shape-based features have been extracted in a number of studies, but not all have retained them in their final radiomic signature. Compactness index has been found to be helpful in differentiating benign from malignant lung nodules^6,7^, to aid in nodule segmentation^8^, and to be associated with distant metastases. In head and neck cancers^9^, the same index has been likewise shown to predict survival^2^ and HPV (Human Papillomavirus) status^10^. Spherical disproportion has been associated with prediction of malignant lung nodules^6^, distant metastases in lung cancers^9^, and HPV status in head and neck cancers^10^. Sphericity was also linked to increased micropapillary component, which portends poorer prognosis, in lung adenocarcinoma^11^. Table 1 summarizes the shape features found significant in publications.

**Table 1:** Shape features found significant in publications.

Shape feature	Author article	Tumor localization	Clinical outcome
Compactness	Aerts^2^, Naturecomms, 2014	Lung cancers, Head and neck cancers	CI of 0.65 (NSCLC) and 0.69 (HNSCC) for survival prediction, with 3 other features (Statistics total energy, GLRL GLN, Wavelet HLH GLN)
	He^8^, IOP Science, 2014	Lung lesions (LIDC-IDRI)	2 features (with average gray value) had CI between computer scores and the reader scores of 0.789 ± 0.014 for nodule subtlety/automatic segmentation
	Wang^6^, IEEE, 2016	Lung lesions (LIDC-IDRI)	Prediction of malignant lung tumor (accuracy: 86% TS, 76% VS) from 15 random forest selected features, 3 of which were shape-based
	Pena^7^, Academic Radiology, 2017	Lung lesions	Prediction of malignant lung tumor with an AUC of 0.92 ± 0.05 (P < 0.0001), with 2 shape features included in a signature of 4 features
	Bogowicz^10^, IJROBP, 2017	Head and neck cancers	Nine features predicted HPV status, including 2 shape features, with AUC = 0.66
	Huynh^9^, PLOS ONE, 2017	Early stage NSCLC (post SBRT)	7 AIP features were associated with distant metastases, 3 of which were shape-based, with CI = 0.648
Spherical Disproportion	Wang^6^, IEEE, 2016	Lung lesions (LIDC-IDRI)	Prediction of malignant lung tumor (accuracy: 86% TS, 76% VS) from 15 random forest selected features, 3 of which were shape-based
	Bogowicz^10^, IJROBP, 2017	Head and neck cancers	Nine features predicted HPV status, including 2 shape features with AUC = 0.66
	Huynh^9^, PLOS ONE, 2017	Early stage NSCLC (post SBRT)	7 AIP features were associated with distant metastases, 3 of which were shape-based, with CI = 0.648
Sphericity	Huynh^9^, PLOS ONE, 2017	Early stage NSCLC (post SBRT)	7 AIP features were associated with distant metastases, 3 of which were shape-based, with CI = 0.648
	Song^11^, IASLC, 2017	LADC	3 features, one of which was shape-based, were predictors of > 5% micropapillary component in LADCs with AUC = 0.61
Surface-To- Volume	Wang^6^, IEEE, 2016	Lung lesions (LIDC-IDRI)	Prediction of malignant lung tumor (accuracy 86% TS, 76% VS) from 15 Random Forest selected features, 3 of which were shape-based
Surface Area	Chaddad^28^, Oncotarget, 2017	NSCLC (TCIA)	Surface area was correlated with the survival time of patients with large cell carcinoma, T2, N0 and Stage I tumors with p < 0.05
S1 (Max. Thickness of The Lesion Skeleton) in 2d	Pena^7^, Academic Radiology, 2017	Lung lesions	Prediction of malignant lung tumor AUC = 0.92 ± 0.05 (P < 0.0001), with 2 shape features included in a signature of 4

NSCLC: non-small cell lung cancer, HNSCC: head and neck squamous cell carcinoma, CI: concordance index, GLRL: gray level run length, GLN: gray level non-uniformity, LIDC-IDRI: Lung Image Database Consortium, SBRT: stereotactic body radiotherapy, AIP: average intensity projection, LADC: Lung adenocarcinoma, TS: Training Set, VS: Validation Set, TCIA: The Cancer Imaging Archive, AUC: area under the curve, HPV: Human Papilloma Virus.

Radiomic features often suffer from being highly correlated, either with tumor volume or with each other, making some of them redundant^12,13^. Too many features, compared to sample size, result in high false discovery rates, over-fitting, and decreased generalizability^1,14,15^. A recent study has shown that certain radiomic features extracted from CT scans, including a shape-based one (compactness), have a high dependency on tumor volume, with Spearman rank correlation coefficients ranging from 0.71 to 0.98^16^.

Moreover, with the field still developing, standards with regards to feature extraction or selection are few and not universally accepted. Because of this, feature nomenclature is not homogenous among publications. A specific example is compactness, which describes how much the shape of a tumor resembles that of a sphere, which has at least three different formulas in the literature^8,17^. Published studies likewise suffer from lack of standardization, making reproducibility of the results a challenge. To tackle these issues, a list of formulas for radiomic feature calculation was proposed by the Image Biomarker Standardization Initiative^18^.

Although different types of medical images such as MRI^19^ or Positron-Emission Tomography (PET) scan^20^ can be analyzed for shape characterization, the use of CT imaging has dramatically increased in the past years^21,22^ leading to numerous shape-based studies using this modality. However, datasets are usually retrospective with a wide range of imaging equipment, acquisition techniques and reconstruction parameters used^23^. Several publications have focused on the influence of these technical aspects on radiomic features (Table 2). A phantom study showed the significant impact of slice thickness on textural features^24^, although shape features generally exhibit more stability^13^. In lung nodules, feature extraction from seven different centers showed that 68% of shape features are robust to segmentation, with concordance correlation coefficients (CCC) > 75%^25^. Another multicentric study showed that shape features were repeatable on test-retest CTs, with standard deviations of 3 to 11%^26^. By adding uncorrelated noise to original images, shape descriptors were however shown to vary more importantly in CT than in PET imaging, with values of 13% and 4%, respectively^27^.

**Table 2:** Radiomic articles on methodology, detailing effects of different acquisition and reconstruction parameters on shape features.

Author/title	Cancer site	Images used for analysis	Radiomics shape features	Other feature classes included	Results
Zhao, 2014^24^ Exploring Variability in CT Characterization of Tumors: A Preliminary Phantom Study	Thorax phantoms with 22 lesions of varying sizes, shapes and densities	1.25, 2.5 and 5 mm slice thickness, Lung and standard reconstruction filters	Compactness, shape index 9 (proportion of the “spherical cap” of the nine types of shapes), fractal dimension, fractal lacunarity	First order statistics and texture features	All 14 features were significantly different between images with 1.25 and 5 mm slice thickness
Kalpathy-Cramer, 2016^25^ Radiomics of Lung Nodules: A Multi-Institutional Study of Robustness and Agreement of Quantitative Imaging Features	Lung nodules	40 NSCLC and 12 phantoms with 9 different segmentations each	7 different centers with varying definitions and number of extracted features including the categories: global shape descriptors, local shape descriptors, margins	First order statistics and texture features	68% of the total 830 features (and 63% of shape features) exhibit stability to different segmentations with CCC ≥ 0.75
Lu, 2016^13^ Assessing Agreement between Radiomic Features Computed for Multiple CT Imaging Settings	32 NSCLC patients (raw imaging data from RIDER dataset)	Varying slice thicknesses (1.25, 2.5 and 5 mm) and reconstruction filter (Lung [L] and Standard [S])	Compact-Factor, Eccentricity, Round-Factor (2D), Solidity (ratio of the object area over the area of the convex hull bounding the object), Shape Index features capturing the intuitive notion of ‘local surface shape’ of a 3D object (spherical cup, trough, rut, saddle rut, saddle, saddle ridge, ridge, dome, spherical cap)	First order statistics, texture and wavelet features	Hierarchical clustering grouped 89 features to 23 nonredundant groups. Majority of the shape-based features showed stability with average CCC values* > *0.8 across all of the 15 inter-setting comparisons. Using the same reconstruction filter with either a 1.25 or a 2.5 mm slice thickness showed the best agreement
Desseroit, 2017^26^ Reliability of PET/CT shape and heterogeneity features in functional and morphological components of NSCLC tumors: a repeatability analysis in a prospective multi-center cohort	Stage IIIB-IV NSCLC Merck MK-0646-008 (40 pts in 17 sites); ACRIN 6678 (34 pts in 14 sites) trials	71 primary tumors and 5 additional lesions	Four shape descriptors: sphericity, irregularity, major axis, 3D surface	First order statistics and texture features	Quantization/discretization was important in the reliability of features, with CT-based features more stable with fixed bin width. Morphological irregularity, sphericity and 3D surface were the most repeatable (Bland-Altman analysis of the differences between standard deviations of 3.3%, 10.0% and 11.6%, respectively)
Oliver, 2017^27^ Sensitivity of Image Features to Noise in Conventional and Respiratory-Gated PET/CT Images of Lung Cancer: Uncorrelated Noise Effects	31 NSCLC patients	4 image sets per patient (original, low, medium, and high noise for 3D & 4D PET, 3D & 4D CT)	11 shape features: Volume, Surface area, Surface-to-volume, Sphericity, Compactness Spherical disproportion, Long axis, Short axis, Eccentricity, Convexity	22 first order, 26 GLCM, 11 GLRLM, and 11 GLSZM features	In both PET and CT, shape features exhibit the least change when uncorrelated noise is added (<13% average difference in CT)
Ul-Hassan, 2017^31^ Intrinsic dependencies of CT radiomic features on voxel size and number of gray levels	ABS 3D printed phantoms, with a spherical contoured ROI of 4.2 cm^3^	116 CT scans, resampled to 1 × 1 × 2 mm^3^ voxel size	10 shape features: Convexity, Volume, Surface area, Surface-to-volume, Compactness, Long axis, Sphericity, Spherical disproportion, Short axis, Eccentricity	First order statistics(16), GLCM (24), GLZSM (11), fractal dimensions, texture and wavelet features	Shape features are robust, with eight out of the 10 having COVs < 50% with a negligible effect of resampling. The remaining two had diminished COV (<30%) after resampling

LoG: Laplacian of Gaussian; NSCLC: Non-small cell lung cancer; CCC: concordance correlation coefficient; GLCM: Gray-Level Co-occurrence Matrix; GLZSM: Gray-Level Size Zone Matrix; GLRLM: Gray-Level Run Length Matrix; SD: standard deviation; ACRIN: American College of Radiology Imaging Network; RIDER: Reference Image Database to Evaluate Therapy Response; ABS: Acrylonitrile Butadiene Styrene; COV: coefficient of variation.

Thus, the aims of this study were (i) to create mathematical models of tumors with increasing degrees of spiculatedness/complexity, (ii) to extract radiomic shape features and determine their relationship with spiculatedness, and (iii) to evaluate the impact of slice thickness, resampling, algorithm used for surface and volume calculations, and change in volume on these features. The ultimate goal is to identify shape features that are least affected by technical parameters such as slice thickness, resampling, and volume, and thus may be prioritized in future radiomic studies.

## Results

The validity of the use of spherical harmonics to model the tumor complexity was verified by asking five different operators to independently classify the forms in order of spiculatedness based on their visual assessment. They were able to classify the tumors correctly, with a maximum error of five forms for one participant, which were consecutive models (*d* = 47 and 50, 86, 89 and 92).

### Comparison of features computed directly from stl models versus after 3d printing, acquisition, segmentation and meshing

**Figure 1:**
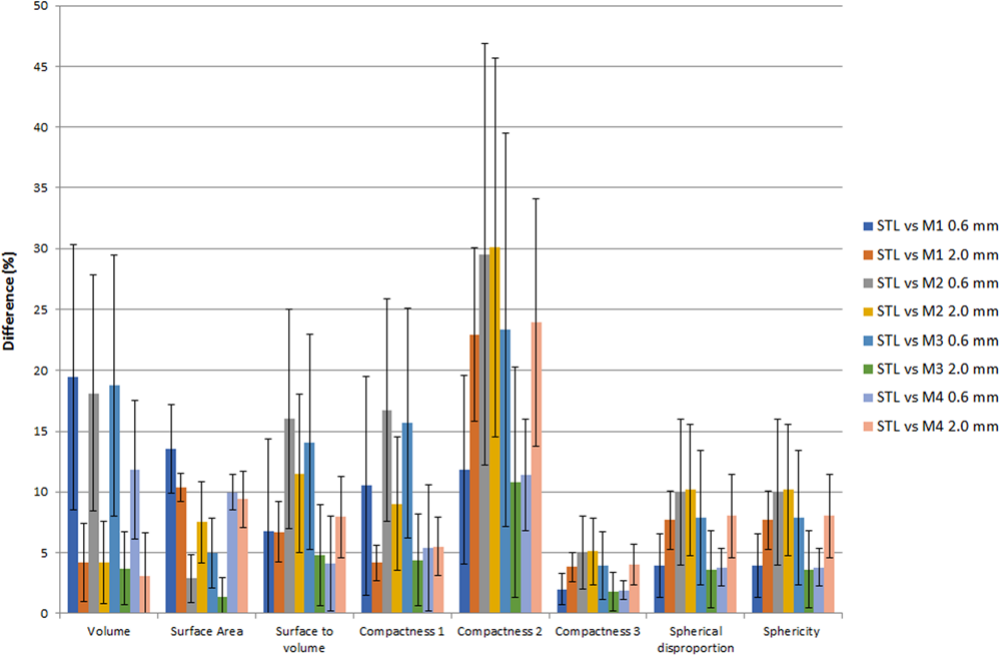
Relative differences between reference shape feature values computed from STL format compared with shape features evaluated after the whole radiomics process including 3D printing, acquisition, image segmentation, and meshing. M1, M2, M3 and M4 meshing methods as well as two slice thicknesses are illustrated here for comparison.

Compared to the 2 mm, the 0.6 mm slice thickness results to increased differences for the feature Volume for all four meshing methods, as seen in Fig. 1. For the 2 mm slice thickness, the differences between the STL derived and the post-processing shape features are lower for the M3 meshing method with mean differences equal to 4.8% (1.0–14.8%, Surface-to-volume), 4.4% (0.7–14.0%, Compactness1), 10.8% (1.5–36.5%, Compactness2), 1.8% (0.3–6.2%, Compactness3), 3.6% (0.5–12.3%, Spherical Disproportion), 3.6% (0.5–12.3%, Sphericity). Standard deviations are slightly decreased for the M1 meshing method compared to M2 and M3 for all features, with values ranging from 1.2 (Compactness3) to 7.1 (Compactness2) and 1.6 (Compactness3) to 9.5 (Compactness2), for the M1 and M3 methods, respectively, when the slice thickness is equal to 2 mm. Standard deviations are lower for the 2 mm slice thickness compared to the 0.6 mm slice thickness, with values ranging from 2.8 (Compactness3) to 16.2 (Compactness2) and 1.6 (Compactness3) to 9.5 (Compactness2), for the 0.6 mm and 2 mm slice thicknesses of the M3 method, respectively.

### Impact of slice thickness on feature values

The nine shape features were first extracted from native binary masks obtained from 2 mm and 0.6 mm slice thickness CT images without any pixel size modification. Change of the slice thickness during the acquisition process leads to mean differences equal to 12.3% (Surface-to-volume, 6.2–26.6%), 13.4% (Compactness1, 5.6–29.1%), 21.4% (Compactness2, 13.1–44.9%), 3.6% (Compactness3, 2.2–7.6%), 7.2% (Spherical Disproportion, 4.4–15.2%), 7.2% (Sphericity, 4.4–15.2%) and 2.6% (0.1–7.5%, Fractional Concavity) for the M1 meshing method. Values were equal to 9.3% (Surface-to-volume, 2.8–23.3%), 11.3% (Compactness1, 3.2–26.9%), 12.7% (Compactness2, 4.1–35.2%), 2.1% (Compactness3, 0.7–5.9%), 4.2% (Spherical Disproportion, 1.3–11.8%), 4.2% (Sphericity, 1.3–11.8%) and 3.0% (0.1–6.1%, Fractional Concavity) for the M3 meshing method.

### Impact of the meshing algorithm on feature values

**Figure 2:**
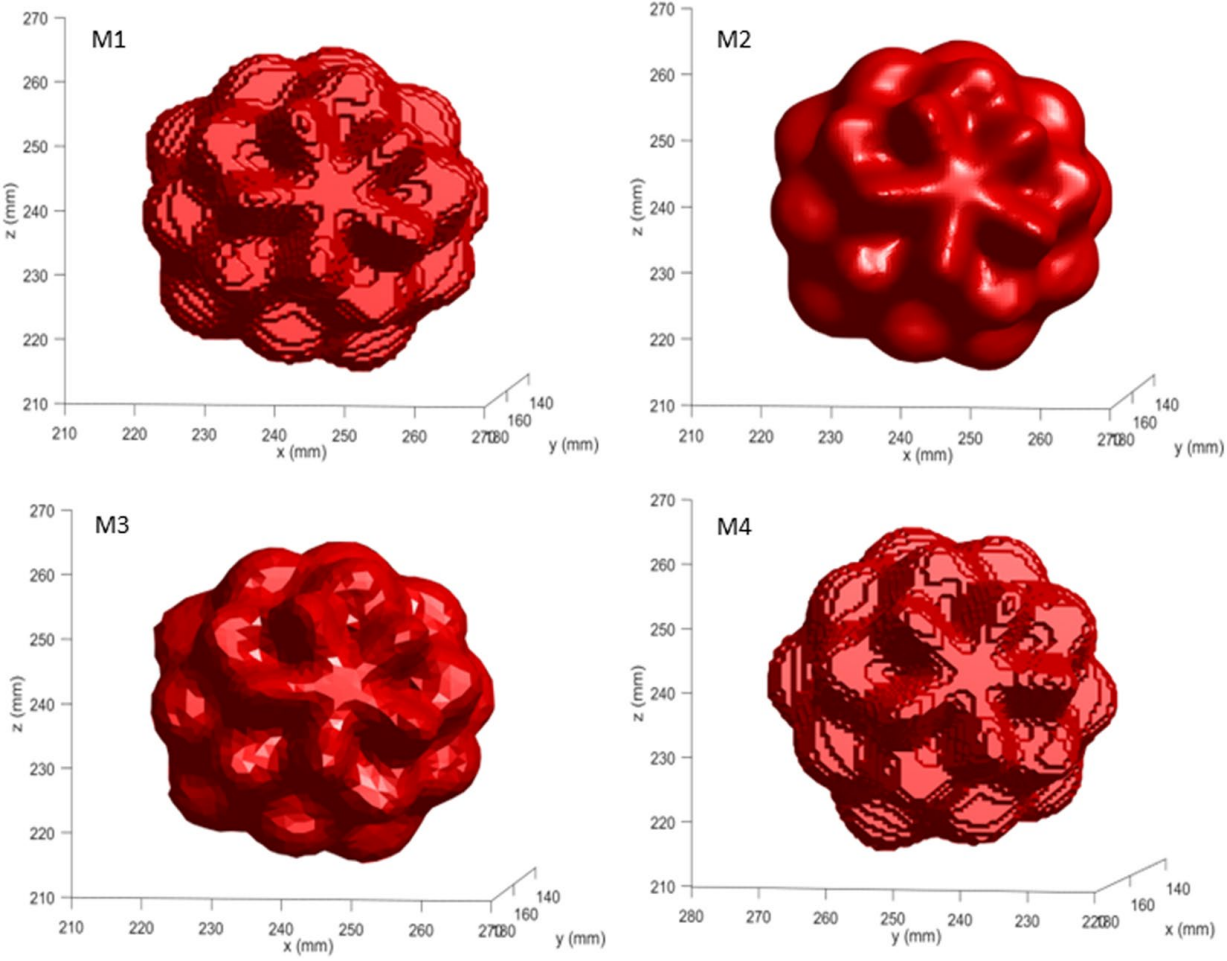
Representation of the meshes obtained for the *d* = 47 tumor model using the M1, M2, M3 and M4 meshing algorithms. CT-images acquired with a 0.6 mm slice thickness were used to extract the binary masks.

**Figure 3:**
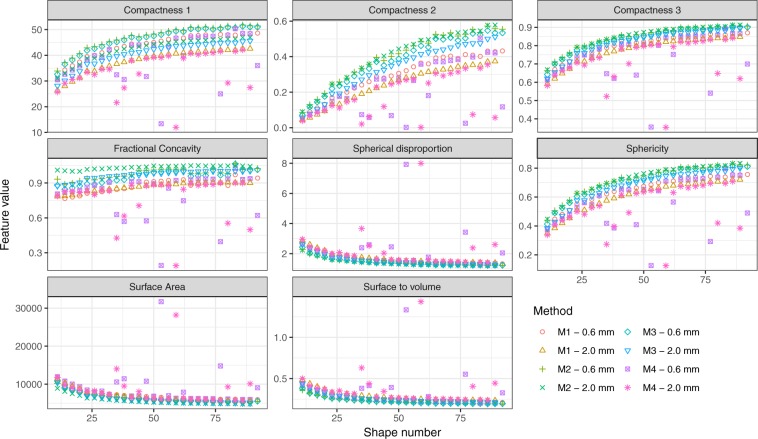
Variation of radiomic shape features as a function of slice thickness, tumor spiculatedness and meshing algorithm used for surface and volume calculation.

Pixel dimensions varied from 0.643 mm to 0.835 mm (X and Y directions) and from 0.565 to 0.733 mm (Z direction) after volume equalization for the masks extracted from the 0.6 mm slice thickness images. These values ranged between 0.639 mm and 0.840 mm (X and Y directions) and between 1.870 to 2.459 mm (Z direction) for the masks extracted from the 2 mm slice thickness images. Figure 2 shows the meshes obtained from the 0.6 mm slice thickness acquisitions for a representative model *d* = 47 for the four algorithms. Figure 3 illustrates the impact of the meshing algorithm and slice thickness on feature values for all the 28 tumor models after volume equalization. Outliers are observed using the Boundary M4 method. Differences ranged from 4.1% (Compactness3, *d* = 77) to 43.2% (Compactness2, *d* = 11) between M1 and M2 methods. Surface area, Surface-to-volume, and Spherical Disproportion had direct relationships with spiculatedness (increasing value with increasing tumor spiculatedness). The three formulas for Compactness, Sphericity and Fractional Concavity had inverse relationships. All features exhibit large variations between *d* = 11 to *d* = 41. Compactness2 is able to highlight shape differences even for the least spiculated models. For this feature, no slope breaking is observed until *d* = 65 irrespective of the slice thickness and meshing method. Subsequent analyses were performed on the 2 mm slice thickness acquisitions, as this is more coherent with what is used in the clinics.

### Correlations between tumor complexity and features

Spearman’s rho correlation coefficients between each shape feature and tumor complexity were computed for the M1, M2 and M3 meshing algorithms (Table 3). Seven of the eight features had strong significant correlations with tumor model complexity irrespective of the meshing algorithm (r > 0.98, p < 10^-4^). Fractional Concavity showed the lowest correlation coefficient (r = 0.83, p < 10^-4^, M2).

**Table 3:** Spearman’s correlation coefficients evaluating the relationship of each shape feature with tumor complexity.

	M1		M2		M3	
	r (95% CI)	p-value*	r (95% CI)	p-value*	r (95% CI)	p-value*
Surface Area	−0.98 (−0.992–0.960)	p < 10^-4^	−0.98 (−0.990–0.952)	p < 10^-4^	−0.98 (−0.992–0.962)	p < 10^-4^
Surface-to-Volume	−0.98 (−0.992–0.960)	p < 10^-4^	−0.98 (−0.990–0.952)	p < 10^-4^	−0.98 (−0.992–0.962)	p < 10^-4^
Compactness1	0.98 (0.960–0.991)	p < 10^-4^	0.98 (0.952–0.990)	p < 10^-4^	0.98 (0.961–0.992)	p < 10^-4^
Compactness2	0.98 (0.960–0.991)	p < 10^-4^	0.98 (0.952–0.990)	p < 10^-4^	0.98 (0.961–0.992)	p < 10^-4^
Compactness3	0.98 (0.960–0.991)	p < 10^-4^	0.98 (0.952–0.990)	p < 10^-4^	0.98 (0.961–0.992)	p < 10^-4^
Spherical Disproportion	−0.98 (−0.992–0.960)	p < 10^-4^	−0.98 (−0.990–0.952)	p < 10^-4^	−0.98 (−0.992–0.962)	p < 10^-4^
Sphericity	0.98 (0.960–0.991)	p < 10^-4^	0.98 (0.952 –0.990)	p < 10^-4^	0.98 (0.961–0.992)	p < 10^-4^
Fractional Concavity	0.94 (0.882–0.975)	p < 10^-4^	0.83 (0.661–0.921)	p < 10^-4^	0.96 (0.920–0.983)	p < 10^-4^

*p-values: Surface Area, Surface-to-Volume, Compactness 1, Compactness 2, Compactness 3, Spherical Disproportion, Sphericity M1: 2.2.10^-16^, M2: 2.2.10^-16^, M3: 2.2.10^-16^, Fractional Concavity M1: 1.2.10^-13^, M2: 7.44.10^-8^, M3: 8.4.10^-16^.

### Correlations between shape features

Correlations among the eight shape features, with the exception of volume that was fixed at a constant value, were calculated using Spearman’s rho coefficients for the M1, M2 and M3 meshing algorithms. Almost all of the features were highly correlated with each other with r = 1, as seen in the correlation matrix plots (Supplementary Figure S1). Only Fractional Concavity was slightly less correlated with the others, with r values from 0.85 to 0.99, with M2 having the lowest correlation.

### Effect of grid resampling

**Figure 4:**
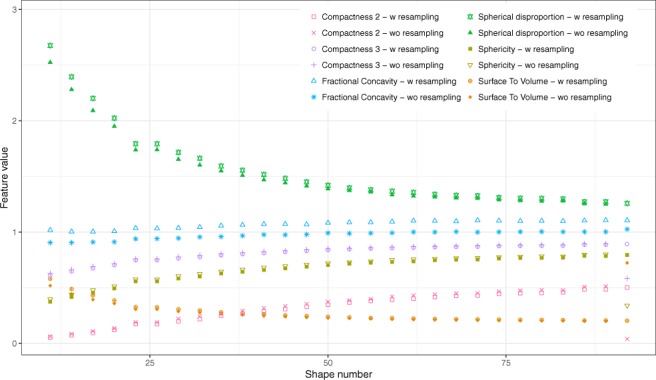
Comparison of feature values extracted from 2 mm thickness original images for each model without and with resampling. Resampling was performed on a 1 × 1 × 1 mm^3^ grid. M3 method was used for feature extraction.

All of the feature values changed when resampling on a 1 × 1 × 1 mm^3^ grid was performed on native masks deduced from the 2 mm thickness original CT images (M1 and M3 meshing methods, no volume equalization). Absolute mean percent changes from the 28 tumor models were equal to 7.8% (5.5–15.7%) for Volume, 7.8% for Surface Area (7.1–10.7%), 1.4% for Surface-to-volume (0.0–5.0%), 2.7% for Compactness1 (0.7–8.6%), 7.5% for Compactness2 (0.6–10.8%), 1.3% for Compactness3 (0.1–1.8%), 2.6% for Spherical Disproportion (0.2–3.6%), 2.6% for Sphericity (0.2–3.6%) and 14.7% for Fractional Concavity (13.8–17.4%) for M1 (Fig. 4) and 7.9% (5.7–16.0%) for Volume, 2.6% for Surface Area (2.0–4.9%), 5.3% for Surface-to-volume (3.2–11.2%), 6.2% for Compactness1 (4.1–12.8%), 8.0% for Compactness2 (4.1–17.4%), 1.3% for Compactness3 (0.7–2.9%), 2.6% for Spherical Disproportion (1.4–5.8%), 2.6% for Sphericity (1.3–5.8%) and 9.5% for Fractional Concavity (8.8–11.6%) for M3 (Figure 4). The ranking of the models was not influenced by the resampling.

### Impact of change in volume on shape features

**Figure 5:**
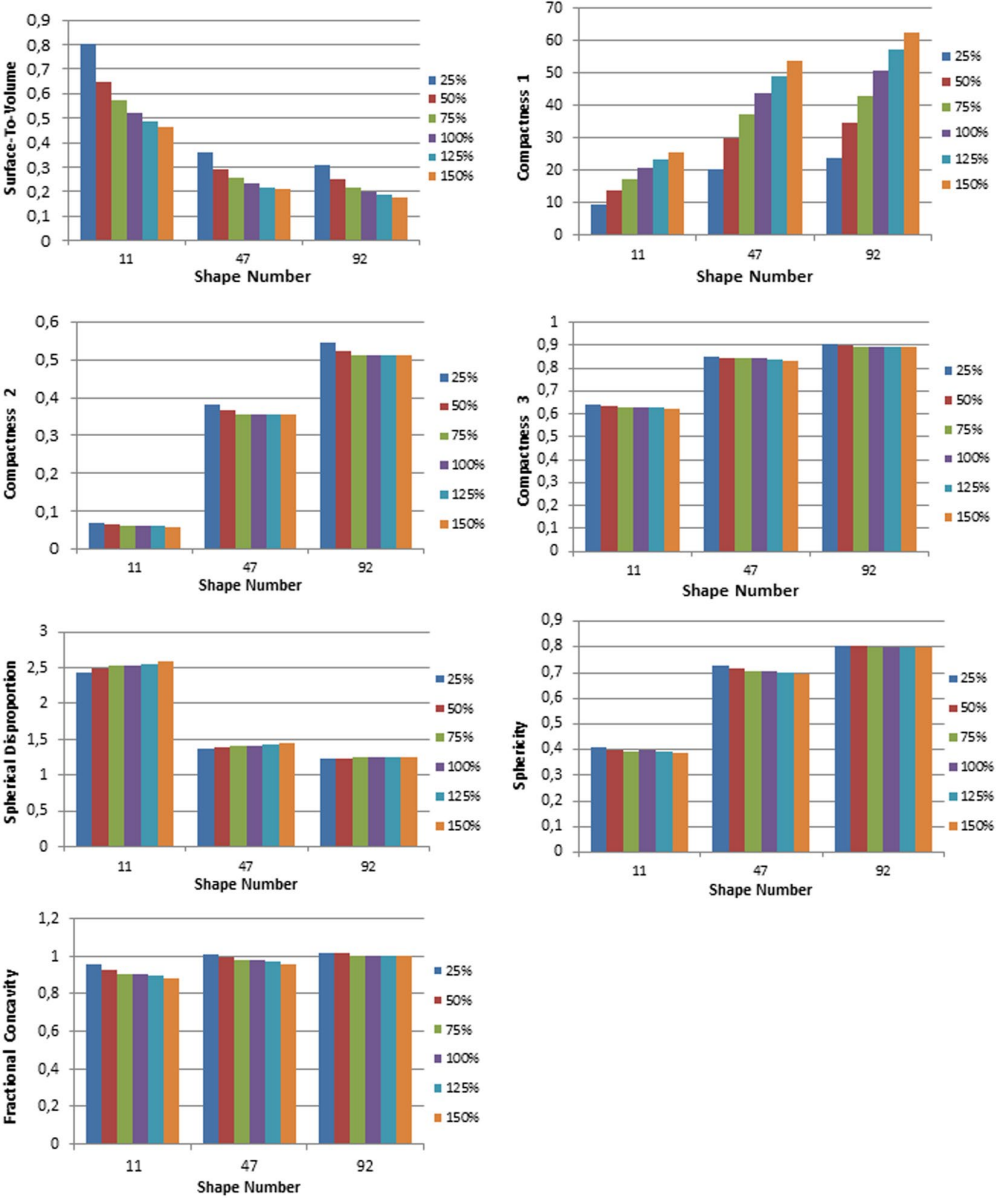
Graphs depicting the change in feature value for changes in volume of 25, 50, 75, 100, 125, and 150% for the three representative features *d* = 11, 47, 92 (M3).

The absolute percent changes of each feature with regards to the change in volume from 25% to 150% were compared to the percent change observed when modifying the spiculatedness of the model from *d* = 11 to *d* = 92 for M3 (Table 4). Compactness2, Compactness3, Spherical Disproportion, Sphericity and Fractional Concavity were less affected by volume changes. Surface-to-volume and Compactness1 were more affected by change in volume than tumor complexity, with the Surface-to-volume feature having a 69.9% change from the least to most spiculated models versus 54.2% change with volume; and Compactness1 having a 47.5% change with spiculatedness compared to 90.4% change with volume. Figure 5 illustrates the change in feature values with changes in volume for the three representative phantoms *d* = 11, 47, 92.

**Table 4:** Absolute percent changes in shape feature values between the most (*d* = 11) and least spiculated models (*d* = 92, first column), and with change in volume of 25% to 150% for the 3 representative models (*d* = 11, 47, 92) obtained for the M3 meshing algorithm.

	*d* = 11 to 92	*d* = 11	*d* = 47	*d* = 92
Surface-to-volume	69.9%	53.9%	54.0%	54.6%
Compactness1	47.5%	90.7%	90.0%	90.3%
Compactness2	160.0%	17.4%	7.8%	6.4%
Compactness3	36.2%	2.9%	2.3%	1.1%
Spherical disproportion	70.2%	5.8%	4.7%	0.8%
Sphericity	70.2%	5.8%	4.7%	0.8%
Fractional concavity	14.8%	8.4%	5.4%	1.1%

**Figure 6:**
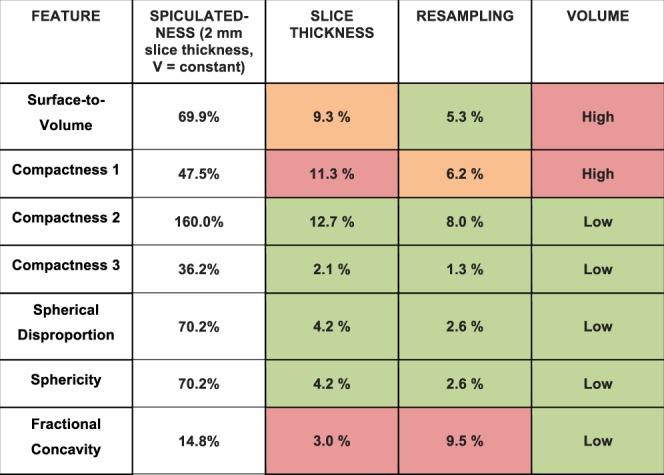
Summary of the effect of technical parameters on the radiomic shape features (M3 meshing method). Effects of the different parameters were compared to the ability of each feature to distinguish change in spiculatedness. Green cases correspond to a ratio of less than 5% between the effect of the technical parameter to the percent change observed when modifying the spiculatedness of the model from *d* = 11 to *d* = 92. Orange cases correspond to ratios ranging from 10 to 20% and red cases to ratios superior to 20%.

Figure 6 summarizes the effect of the technical parameters slice thickness, resampling, and change in volume on the radiomic shape features, reiterating that Compactness1 has important variations with changes both in slice thickness and in volume, and Surface-to-volume with change in volume.

### Comparison with actual tumors

To confirm the clinical relevance and applicability of our method, three representative patients from the RIDER database were contoured, and radiomic shape features were extracted thereafter. The range of values for each of the features among the three patients are within the range of the values from the shape phantoms, even if the volume after equalization was greater in the phantoms (range: 28257–28265 mm^3^). Patient volumes ranged from 5890 mm^3^ to 21045 mm^3^. The results for the three patients as well as the mean values for the phantoms are shown in the Supplementary Table S2.

## Discussion

This is the first methodological study that directly demonstrates the relationship of tumor spiculatedness with radiomic shape features. Models with increasing degrees of spiculatedness were created to examine the behavior of quantitative shape features with known incremental degrees of tumor border complexity. It was seen that specific features increase monotonically with increasing tumor spiculatedness, in particular Surface Area, Surface-to-volume, and Spherical Disproportion. Conversely, certain features exhibit a monotone decreasing correlation with increasing spiculatedness (Compactness, Sphericity, Fractional Concavity). Quantitative extracted shape features have already been demonstrated to give insights on tumor behavior, underlying their importance in radiomic analysis. Based on CT scans, several publications have shown that shape features differentiate between benign and malignant nodules^6–8^ as well as correlate with patient outcomes^2,9,28^. In addition, a radiomic study of pre-treatment contrast-enhanced T1 MRI images in glioblastoma showed that tumor surface regularity was a powerful predictor of survival in the discovery (*p* = 0.005, hazard ratio [HR] = 1.61) and validation groups (*p* = 0.05, HR = 1.84)^29^.

It can be seen that many of the features exhibit strong correlations with each other, either positive or negative. If the behaviors of certain features are known to depend on specific parameters, calculating all may not necessarily give complementary information but instead redundant ones. In particular, Surface-to-volume, Compactness, Spherical Disproportion and Sphericity are all calculated from tumor volume and surface area^17^, which explains the strong relationships among them. In this regard, features might eventually be grouped into clusters instead of being analyzed individually^12^. For instance, different formulas for compactness have been previously published and used in radiomic studies^8,17^. In clinical studies, it needs to be determined whether correlations seen with radiomic shape features are inherent, or if tumor volume is a confounding factor. In our study, it is seen that Surface-to-volume and Compactness1 are affected with volume changes, and should thus be used with caution when comparing tumors with differing volumes. Indeed, even if it has been widely used in previous publications, the result obtained from the formula for Compactness1 is not dimensionless, and thus is not ideal in feature analysis. Compactness2, Compactness3, Spherical Disproportion, and Sphericity’s percent changes between *d* = 11 and *d* = 92 were noticeably higher than the percent change with volume variations, which may make these features more useful in analyses of patient tumors as they are not volume dependent. In addition, it was also seen that in general, the features highlight differences in complexity better in more spiculated tumors. For instance, in this study, the slope of the relationship between feature and spiculatedness was steep until *d* = 41, and thereafter relatively flattened out for the less spiculated models.

In this study, four meshing algorithms have been used and the differences between the features computed directly from the STL files and those computed after meshing have been compared (Fig. 1). Values extracted using M3 meshing method are the closest to the STL reference ones, with mean differences lower than 10.8% (Compactness2) for all features. The decrease of the standard deviation when comparing the values from STL files versus from M1 and M3 with 0.6 or 2 mm slice thickness (Fig. 1) validates the fact that a 2 mm slice thickness should be preferred for shape-based radiomic analysis. With a 2 mm slice thickness, shape features are impacted in a more homogeneous way by the entire radiomics process. The associations of the deduced shape features with change in tumor complexity have also been analyzed for each method (Fig. 3). Using the MATLAB Boundary function, a non-monotone behavior of the features with phantom spiculatedness was observed, with the presence of outliers. The use of this function is thus not recommended in in-house MATLAB-based softwares. Comparison of the M1, M2 and M3 methods shows that different meshing implementations can lead to different quantitative values. As a consequence, thresholds determined in the literature should be used with caution. Numerical phantoms such as the ones developed in this study can be also of major interest for the evaluation of the pertinence of meshing algorithms as well as for the development of new shape features. Notable is that in this study, we chose to use meshing for volume extraction, which is not performed in most of the radiomic software that typically multiplies the voxel size by the number of voxels in the volume of interest^30^. This choice is of importance for maintaining consistency between surface and volume quantities.

Comparisons of feature values between scans acquired with 0.6 mm versus 2 mm slice thickness reveal that this parameter affects all radiomic shape features, with changes of up to 12.7% (Compactness1, M3). Resampling the CT images on a 1 × 1 × 1 mm^3^ grid likewise resulted in small differences of between 1.3 to 9.6% changes in extracted features for the isosurface remesher M3 method. In a phantom study that computed the differences between original features and those resampled on a 1 × 1 × 2 mm^3^ grid with original pixel sizes ranging from 0.39 to 0.98 mm, shape features belonged to the group that were generally not significantly affected by resampling^31^. In this study, the Credence Cartridge Radiomics (CCR) phantoms used were rectangular in shape and created primarily for texture analysis, whereas ours had fine spiculations specifically created for shape analysis. Another study using the same phantoms showed that radiomic features were affected by slice thickness, but that this effect could be reduced by resampling the images before feature extraction. However, this study focused on 114 first order and textural features and did not include shape^32^. In yet another phantom study using spherical, elliptical, lobulated and spiculated forms, it was shown that shape features were significantly different between 1.25 and 5 mm slice thickness scans^24^, from which we can infer that voxel size affects results of feature extraction. At present, we therefore recommend not to constitute a cohort with images having too different slice thicknesses particularly if the Compactness1, Surface-To-Volume and Fractional Concavity indices are computed, given their dependence on slice thickness. However, the ideal is prospective studies with homogenous acquisition parameters, as resampling alone does not completely eliminate bias resulting from differences in acquisition such as slice thickness.

There are disadvantages to this study. First, only 3D features were calculated as the tumor models were contoured on axial CT slices and had discontinuous islets on some slices (usually at the top and edges of the tumor) because of the spiculations. In addition, the tumor phantoms were printed with a flat base, instead of a spherical-based shape with no flat edges due to technical considerations for 3D printing. However, all the phantoms were created in the same manner (with a flat base) such that all shape feature variations are expected to be comparable. Another limitation is that although the shape phantoms have increasing degrees of complexity, the variations of these are all based on the formula of spherical harmonics and thus have a consistent mathematical progression. Actual tumors are rarely symmetric and regularly shaped. However, theoretical knowledge of how radiomic shape features vary remains of value in deducing the complexity of actual tumors. Also, in studying variations with volume, the volumes were modified mathematically by recomputing the pixel sizes, which are inherently correct; but another way would have been to do a 3D reprint of each model with each corresponding volume change. Another limitation in the conduct of radiomics studies in general is that there is no generally accepted and universally utilized meshing method, and as illustrated in this study, different methods do not result to identical values.

In summary, majority of radiomic shape features have strong monotone direct or inverse correlations with tumor spiculatedness. However, we have shown that quantitative values of these features can vary with slice thickness, volume, and resampling; and depend on the meshing algorithm used for surface and volume extraction. The radiomic shape features Compactness2, Compactness3, Spherical Disproportion, and Sphericity have been shown to have minimal variations with the aforementioned parameters, and should thus be prioritized in future studies. It is clear that quantitative radiomic shape features provide important information on tumor characteristics, underlining the importance of their integration into future radiomic models and notably their combination with clinical, textural and genomic features. Refinements in the methodology of conducting radiomic studies as well as transparency in the exact nomenclature and formula used for each feature are indispensable to enable its eventual translatability to clinical utility.

## Material and Methods

### Shape phantoms

Spherical harmonics were used to create mathematical tumor models with increasing degrees of complexity^28^.

$${Y}_{l}^{m}(\theta ,\phi )=\sqrt{\frac{(2l+1)}{4\pi }\frac{(l-m)!}{(l+m)!}}{P}_{l}^{m}(cos(\theta )).{e}^{im\phi }$$

with *ℓ* the degree, *m* = {*− ℓ*, − *(ℓ −1*)*, …, 0, …, ℓ* − *1, ℓ | ℓ* ∈ ***ℕ***} indicating the order, *θ* and *φ* the polar and azimuthal coordinates and ${P}_{l}^{m}$ an associated Legendre polynomial^33^. Using the real component of ${Y}_{l}^{m}(\theta ,\phi )$ cartesian coordinates x, y and z were deduced as follows:

$$x=\rho .\,\sin (\theta ).\,\cos (\phi )$$

$$y=\rho .\,\sin (\theta ).\,\sin (\phi )$$

$$z=\rho .\,\cos (\theta )$$

with

$$\rho =d+\frac{A.Re\{{Y}_{l}^{m}(\theta ,\phi )\}}{{\rm{\max }}(abs(Re\{{Y}_{l}^{m}(\theta ,\phi )\}))}$$

In the presented work, *ℓ* was arbitrarily set to 10, *m* to 5 and A to 10. The degree of the spherical harmonic, *d*, was increased in increments of 3 from 11 to 92 to create a total of 28 models. Model “11” corresponded to the most spiculated model and model “92” to the least one.

The 3D models were cut in the middle of the horizontal plane which permitted these to have a flat base for printing. Then, models were set with identical bases of 5 cm by adjusting the height ratio of the original models to the new base. Each of the tumor models was created using a 3D printer (Discoeasy200, *dagoma.fr*), using a polylactic acid filament (ρ = 1.25 g.cm^−3^) with standard printing speed (Supplementary Figure S3).

The models were then scanned using a Siemens Sensation Open CT scan (Siemens Healthineers, Erlangen, Germany) with 0.6 mm and 2 mm slice thicknesses, 100 kVp tube voltage, 300 mAs and a 350 mm reconstruction field of view. The phantoms were scanned on top of a cardboard box, with only the bases being in contact with a surface. Original pixel size was 0.68 mm × 0.68 mm in the transverse planes. Scans were contoured using the thresholding function of 3DSlicer (http://www.slicer.org), with lower limit at −700 Hounsfield Units (HU) and upper limit at 3000 HU, resulting in binary masks.

### Feature extraction

Four different approaches based on MATLAB libraries (R2017b software (The Mathworks Inc., Natick, MA, USA) were used to generate a mesh for all tumor models, which involved creating a surface mask from the contours (Supplementary Table S4). The first method (M1) used the Isosurface function of MATLAB. This method connects points having the same value to generate the mask. The isovalue was set to 0.9. The second method (M2), Isosurface filter, consisted of smoothing the triangulated mesh generated with the first method by using the normalized curvature operators as weights. The mesh was mainly smoothed in the normal direction to preserve the original ratio in length between edges. One smoothing iteration was used and the smoothing quantity was set to 5. The third strategy (M3), Isosurface remesher, is an iterative triangle optimization for meshing. In this method, all the closed meshes obtained with the first method are cleaned according to a targeted edge length. The edge length was set to 2 and only one iteration was used. The last method (M4) used the Boundary function of MATLAB that returns a triangulation corresponding to a single conforming 3D boundary around the points. A shrinking factor of 1 was used to obtain the concave hull of the shape of interest.

Nine three-dimensional (3D) shape features were deduced from the surface and volume values extracted using the four meshing methods previously described. These included Volume, Surface Area, Surface-to-volume, three formulas for Compactness^8^, Spherical Disproportion, Sphericity^17^ and Fractional Concavity^34^. Table 5 shows the description and formulas of the computed features. The surface of the convex hull included in the fractional concavity formula was obtained using the Boundary function and a shrinking value of 0.

**Table 5:** Radiomic shape feature formulas.

Feature	Description	Formula
Volume	Compute the enclosed volume of the object of interest. The enclosed volume is evaluated by triangulation (*i.e*. dividing the surface into connected triangles)	Green-Ostrogradski formula: ${\iiint }_{{\rm{V}}}\overrightarrow{{\boldsymbol{\nabla }}}\overrightarrow{{\rm{F}}}{\rm{.dV}}={\iint }_{{\rm{S}}}\overrightarrow{{\rm{F}}}{\rm{.dS}}$ where F corresponds to the vector field deduced from the triangulation
Surface area^17^	Area of the surface encompassing the volume of interest, calculated by triangulation	$A=\sum _{i=1}^{N}\frac{1}{2}|{a}_{i}{b}_{i}\times {a}_{i}{c}_{i}|$
Surface-to-volume ratio^17^	Ratio of surface to volume	$Surface\,to\,volume\,ratio=\frac{A}{V}$
Compactness1^17^	Describes how much the shape of a tumor resembles that of a sphere/can be encompassed by a sphere Compactness of a sphere = 1	$compactness\,1=\frac{V}{\sqrt{\pi .}{A}^{2/3}}$
Compactness2^17^		$compactness\,2=36.\pi .\frac{{V}^{2}}{{A}^{3}}$
Compactness3^8^		$compactness\,3=\frac{{V}^{1/3}.{(36\pi )}^{1/6}}{\sqrt{A}}$
Spherical disproportion^17^	The ratio of the surface area of the tumor to the surface area of a sphere with the same volume as the tumor	$Spherical\,disproportion=\frac{A}{4\pi {R}^{2}}$
Sphericity^17^	Measure of the roundness or spherical nature of the tumor, where the sphericity of a sphere is the maximum value of 1	$Sphericity=\frac{{(\pi )}^{1/3}{(6V)}^{2/3}}{A}$
Fractional concavity^34^	The ratio between the surface of the convex hull encompassing the tumor, and the actual surface of the tumor.	$Fc{c}_{-}3D=\frac{Surface\,of\,the\,convex\,hull}{A}$

A: area, V: volume, R: radius.

To quantify the impact of 3D printing, acquisition, segmentation and meshing on the radiomic shape features, features were computed directly from the stereolithography (STL) file format that was used for 3D printing, and compared to those computed using the aforementioned Matlab functions.

To study the effect of resampling, masks extracted from the original images were resampled on a 1 × 1 × 1 mm^3^ grid using a 3D linear interpolation. To remove the inherent variation on volume between objects, a homothetic transformation was then applied to bring back all the volumes to the value corresponding to the average of all volume values calculated for the 28 shapes for the M1 method. Finally, three representative phantoms (*d* = 11, 47, 92) were resampled to have 25, 50, 75, 125 and 150% of their original volume.

To validate the clinical relevance of the phantoms, CT-scans of lung tumors of three patients from the publicly available RIDER database^35^ were contoured. Radiomic features were extracted and compared to the range of the values extracted from the 28 printed phantoms.

### Statistical analysis

**Figure 7:**
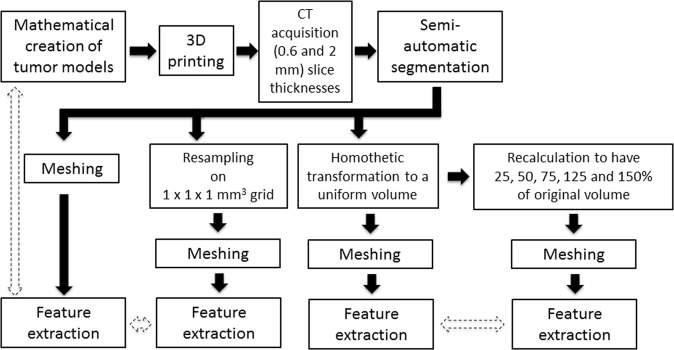
Schema of the steps undertaken in the study. Broken arrows represent comparison between the original extracted features and after resampling or after volume changes.

All statistical analyses were performed with R version 3.3.2 (https://www.r-project.org/). Differences between features computed directly from the STL files and those computed after meshing were compared. Percent changes between scans acquired with 0.6 and 2 mm slice thicknesses were quantified. To determine the relationship of each shape feature with tumor complexity, Spearman’s rank-order correlation coefficients were computed for each of the four meshing methods. Complexity was considered as an ordinal variable with numeric values ranging from 11 (most spiculated) to 92 (least spiculated). Pairwise correlations among variables were also computed. To evaluate the effect of resampling on feature variation, percent changes were computed between features extracted from original and resampled (1 × 1 × 1 mm^3^ grid) 3D masks for the M1 and M3 meshing methods. To evaluate the effect of changes in volume, the percent change in shape features when the volume varied from 25% to 150% was computed for *d* = 11, 47 and 92 and compared to the percent change observed when modifying the spiculatedness of the model from *d* = 11 to *d* = 92 for M3. Figure 7 summarizes the general schema of the methodology.

## Supplementary information

The complexity of tumor shape, spiculatedness, correlates with tumor radiomic shape features.

## Data Availability

The datasets generated during and/or analyzed during the current study are available from the corresponding author on reasonable request.

## References

[1] Gillies RJ, Kinahan PE, Hricak H (2015). Radiomics: Images Are More than Pictures, They Are Data. Radiology.

[2] Aerts HJWL (2014). Decoding tumour phenotype by noninvasive imaging using a quantitative radiomics approach. Nat Commun.

[3] Limkin EJ (2017). Promises and challenges for the implementation of computational medical imaging (radiomics) in oncology. Ann Oncol.

[4] Edge SB, Compton CC (2010). The American Joint Committee on Cancer: the 7th edition of the AJCC cancer staging manual and the future of TNM. Ann Surg Oncol.

[5] Razek AA, Huang BY (2011). Soft tissue tumors of the head and neck: imaging-based review of the WHO classification. Radiographics.

[6] Wang, J., *et al*. Prediction of malignant and benign of lung tumor using a quantitative radiomic method. In: 2016 *38th Annual International Conference of the IEEE Engineering in Medicine and Biology Society (EMBC)*, pp 1272–1275 (2016). 10.1109/EMBC.2016.7590938 28268557

[7] Pena E (2017). Can CT and MR Shape and Textural Features Differentiate Benign Versus Malignant Pleural Lesions?. Acad Radiol.

[8] He X, Sahiner B, Gallas BD, Chen W, Petrick N (2014). Computerized characterization of lung nodule subtlety using thoracic CT images. Phys Med Biol.

[9] Huynh E (2017). Associations of Radiomic Data Extracted from Static and Respiratory-Gated CT Scans with Disease Recurrence in Lung Cancer Patients Treated with SBRT. PLoS One.

[10] Bogowicz M (2017). Computed Tomography Radiomics Predicts HPV Status and Local Tumor Control After Definitive Radiochemotherapy in Head and Neck Squamous Cell Carcinoma. Int J Radiat Oncol Biol Phys.

[11] Song SH (2017). Imaging Phenotyping Using Radiomics to Predict Micropapillary Pattern within Lung Adenocarcinoma. J Thorac Oncol.

[12] Berenguer, R. *et al*. Radiomics of CT Features May Be Nonreproducible and Redundant: Influence of CT Acquisition Parameters. *Radiology*. 172361 (2018). 10.1148/radiol.2018172361 29688159

[13] Lu L, Ehmke RC, Schwartz LH, Zhao B (2016). Assessing Agreement between Radiomic Features Computed for Multiple CT Imaging Settings. PLoS One.

[14] Kumar V (2012). Radiomics: the process and the challenges. Magn Reson Imaging.

[15] Ferté C (2013). Impact of bioinformatic procedures in the development and translation of high-throughput molecular classifiers in oncology. Clin Cancer Res.

[16] Vallieres M, Visvikis D, Hatt M (2018). Dependency of a validated radiomics signature on tumor volume and potential corrections. J Nucl Med.

[17] Coroller TP (2015). CT-based radiomic signature predicts distant metastasis in lung adenocarcinoma. Radiother Oncol.

[18] Zwanenburg, A., Leger, S., Vallières, M. & Löck, S. Others. Image biomarker standardisation initiative-feature definitions. a*rXiv preprint arXiv:1612 07003*. https://arxiv.org/abs/1612.07003 (2016).

[19] Li H (2016). MR Imaging Radiomics Signatures for Predicting the Risk of Breast Cancer Recurrence as Given by Research Versions of MammaPrint, Oncotype DX, and PAM50 Gene Assays. Radiology.

[20] Hatt M (2018). Tumour functional sphericity from PET images: prognostic value in NSCLC and impact of delineation method. Eur J Nucl Med Mol Imaging.

[21] Smith-Bindman R, Miglioretti DL, Larson EB (2008). Rising Use Of Diagnostic Medical Imaging In A Large Integrated Health System. Health Aff.

[22] CT Benchmark Report. M*edical Information Division* (2014).

[23] Mackin D (2015). Measuring Computed Tomography Scanner Variability of Radiomics Features. Invest Radiol.

[24] Zhao B, Tan Y, Tsai WY, Schwartz LH, Lu L (2014). Exploring Variability in CT Characterization of Tumors: A Preliminary Phantom Study. Transl Oncol.

[25] Kalpathy-Cramer J (2016). Radiomics of Lung Nodules: A Multi-Institutional Study of Robustness and Agreement of Quantitative Imaging Features. Tomography.

[26] Desseroit M-C (2017). Reliability of PET/CT Shape and Heterogeneity Features in Functional and Morphologic Components of Non-Small Cell Lung Cancer Tumors: A Repeatability Analysis in a Prospective Multicenter Cohort. J Nucl Med.

[27] Oliver JA (2017). Sensitivity of Image Features to Noise in Conventional and Respiratory-Gated PET/CT Images of Lung Cancer: Uncorrelated Noise Effects. Technol Cancer Res Treat.

[28] Chaddad A, Desrosiers C, Toews M, Abdulkarim B (2017). Predicting survival time of lung cancer patients using radiomic analysis. Oncotarget.

[29] Pérez-Beteta, J. e*t al*. Tumor Surface Regularity at MR Imaging Predicts Survival and Response to Surgery in Patients with Glioblastoma. R*adiology*, 171051 (2018). 10.1148/radiol.2018171051 29924716

[30] Shafiq-Ul-Hassan M (2018). Voxel size and gray level normalization of CT radiomic features in lung cancer. Sci Rep.

[31] Shafiq‐ul‐Hassan, M., Zhang, G. G. & Latifi, K. Intrinsic dependencies of CT radiomic features on voxel size and number of gray levels. *Medical*, https://vpn.igr.fr/doi/10.1002/mp.12123/,DanaInfo=onlinelibrary.wiley.com+full (2017). 10.1002/mp.12123 PMC5462462 28112418

[32] Larue RTHM (2017). Influence of gray level discretization on radiomic feature stability for different CT scanners, tube currents and slice thicknesses: a comprehensive phantom study. Acta Oncol.

[33] De Santis A, Torta JM, Falcone C (1996). A simple approach to the transformation of spherical harmonic models under coordinate system rotation. Geophys J Int.

[34] Rangayyan RM, Nguyen TM (2007). Fractal analysis of contours of breast masses in mammograms. J Digit Imaging.

[35] Armato SG (2008). The Reference Image Database to Evaluate Response to therapy in lung cancer (RIDER) project: a resource for the development of change-analysis software. Clin Pharmacol Ther.

