# Supplementary material for: The complexity of tumor shape, spiculatedness, correlates with tumor radiomic shape features
Source: Sci Rep. 2019 Mar 13;9:4329. doi: 10.1038/s41598-019-40437-5 (PMC6416263; doi:10.1038/s41598-019-40437-5)
Supplement: Supplementary file 1 — The complexity of tumor shape, spiculatedness, correlates with tumor radiomic shape features. [file 41598_2019_40437_MOESM1_ESM.docx]

SUPPLEMENTARY DATA

TITLE:

The complexity of tumor shape, spiculatedness, correlates with tumor radiomics features.

AUTHORS:

Elaine Johanna Limkin^1,2^, Sylvain Reuzé†^2,3,4^, Alexandre Carré†^3,4^, Roger Sun^1,2,3^, Antoine Schernberg^1,2,3^, Anthony Alexis^1,2^, Eric Deutsch††^1,2,3^, Charles Ferté††^2,5^, Charlotte Robert*^2,3,4^

Supplementary Figure S1. Correlation plot among features evaluated by Spearman’s correlation coefficients for M1, M2 and M3 meshing methods


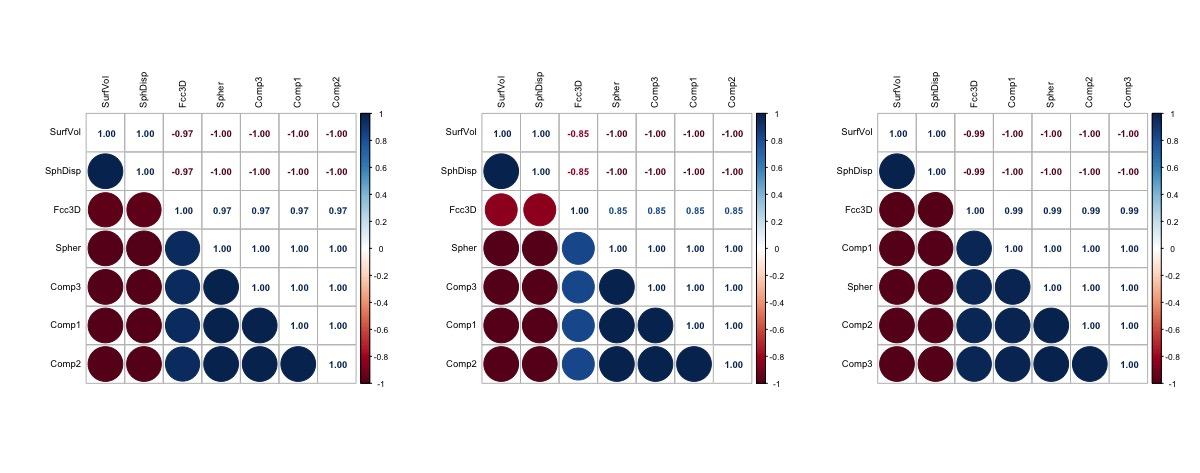


Supplementary Table S2. Radiomic feature values for three representative patients and the range for the shape phantoms using the meshing method M1.

|  | **Phantoms (min-max)** | **Patient 1** | **Patient 2** | **Patient 3** |
| --- | --- | --- | --- | --- |
| **Volume** | 28257.08 - 28265.08 | 21045.31 | 9092.33 | 5890.88 |
| **Surface Area** | 5936.03 - 11816.49 | 5428.08 | 4226.46 | 2403.96 |
| **Surface to volume** | 0.21 - 0.41 | 0.25 | 0.46 | 0.41 |
| **Compactness 1** | 30.73 - 48.63 | 38.44 | 19.62 | 18.52 |
| **Compactness 2** | 0.05 - 0.43 | 0.31 | 0.12 | 0.28 |
| **Compactness 3** | 1.35 - 1.91 | 1.81 | 1.55 | 1.78 |
| **Spherical disproportion** | 1.32 - 2.63 | 1.47 | 2.01 | 1.52 |
| **Sphericity** | 0.37 - 0.75 | 0.68 | 0.50 | 0.66 |
| **Fractional Concavity** | 0.76 - 0.97 | 0.97 | 0.85 | 0.92 |

Supplementary Figure S3. Tumor models with decreasing spiculatedness from bottom left to upper right


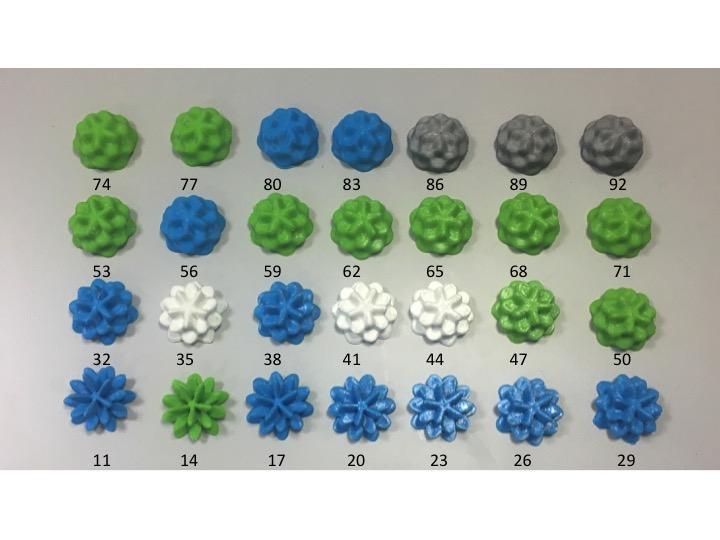


Supplementary Table S4. Description of the 4 MATLAB meshing algorithms used for volume and surface calculations.

|  | **M1 Isosurface** | **M2 Isosurface filter** | **M3**  **Isosurface remesher** | **M4 Boundary** |
| --- | --- | --- | --- | --- |
| **MATLAB function** | isosurface() | isosurface() + smoothpatch() | isosurface() + remesher() | boundary() |
| **Parameters** | isovalue = 0.9 | isovalue = 0.9;  mode = 1;  itt = 1;  lambda = 5;  sigma = 1 | isovalue = 0.9;  edgelength = 2;  iterations = 1 | s = 1 |
